# Epidemiology and genotype distribution of human papillomavirus (HPV) in Southwest China: a cross-sectional five years study in non-vaccinated women

**DOI:** 10.1186/s12985-017-0751-3

**Published:** 2017-04-21

**Authors:** Yishu Tang, Lan Zheng, Shuangshuang Yang, Bing Li, Huiting Su, Li-ping Zhang

**Affiliations:** Department of Laboratory Medicine, The First Affiliated Hospital of Chongqing Medical University, No. 1 Youyi Road, Yuzhong District, 400016 Chongqing, People’s Republic of China

## Abstract

**Background:**

Large-size data on type-specific HPV prevalence in Southwest China are required to estimate the cervical cancer burden in the country and to prepare for HPV-based cervical screening program and further HPV vaccination of China. This HPV study is a pooled analysis of data from five years in Chongqing of China, which is cross-sectional in design using data collecting.

**Results:**

The positivity of HPV was 26.2% (10542/40311), single type was 25.7% (10360/40311), multiple type was 8.2% (3306/40311), high-risk HPV was 30.9% (12490/40311), and low-risk HPV was 2.9%(1169/40311). The most common genotypes were HPV16,52,58 and 18. HPV-positive women (*n* = 10542) were triaged by cytology, colposcopy or histological diagnosis. Among HPV-positive women, 43.8% had normal, 22.5% had ASCUS, 0.2% had LSIL, 12.6% had HSIL and 6.0% had ICC. The most common HPV genotypes were HPV16, 58 and 18 in ASCUS, HPV16, 18 and 58 in LSIL, HPV16, 58 and 33 in HSIL, and HPV16, 58 and 18 in ICC. The prevalence of Group 1/2A HPV types increased with increasing CIN grade and accounted for 96.05% of the CIN 3+ lesions, while HPV16 accounted for 71.1%. HPV-positive women steadily increased with age, peaking at 31–40 years.

**Conclusion:**

The type-specific prevalence rate of HPV 16 and HPV 18 were a little lower than the mean of international meta-analyses. Single HPV genotype infection was predominantly detected in different groups of cervical lesions in Chongqing, and HPV16, 52, 58 were the priority HPV types. The HPV genotyping study was found to be valuable for planning further preventive program for cervical cancer.

## Background

Cervical cancer is the second most common female cancer worldwide, which results in >250000 deaths annually [1]. The infection of Human papillomavirus (HPV) is the most common sexually transmitted disease, which is the major reason of cervical squamous intraepithelial lesions as well as cervical cancer [2, 3]. From the general population it is established that 80% will be infected at some time during their life [4].

More than 100 different HPV genotypes have been identified, of which, 40 infect the genital tract [5]. In general, they are divided into low-risk and high-risk categories according to their ability to induce cervical cancer, and geographic differences [6]. Currently, there are 12 HPV types classified as Group 1 carcinogens and one type that is probably carcinogenic (Group 2A carcinogen) [7]. All of these types belong to the same evolutionary branch in the phylogenetic tree of papillomaviruses, which suggest that the carcinogenicity reflects viral evolution [8]. A larger proportion of screen-detected HPV infections represent persistent HPV infections with increasing age. However, it should be noted that even if some HPV types do persist, they will never cause neoplastic progression [8, 9].

HPV DNA testing has emerged as a worthful complementary method to conventional Papanicolaou (Pap) staining for enhancing the effect of cervical cancer screening. It has played an indispensable role in determining suitable clinical management schemes for cervical cancer screening and subsequent HPV vaccination plan [10–12].

Cervical cancer could be effectively controlled by primary and secondary prevention like cervical screening and prophylactic HPV vaccination. However, to date there are no large-size data on type-specific HPV prevalence in Southwest China women, especially in Chongqing, making it difficult to design a national HPV vaccination strategy. Because government validation for the HPV vaccines will likely be upcoming after the fulfillment of ongoing clinical studies, an extensive understanding of type-specific HPV prevalence among Chinese women is crucial. Data on the type-specific prevalence of carcinogenic HPV types are required to supervise the effect of vaccination and to formulate HPV-based cervical screening strategies of Chinese women across the country when the vaccines are taken. The purpose of the study was to provide valuable data about the type-specific prevalence of HPV infection and its connection to findings in histology and cytology triage.

## Methods

### Study subject recruitment and sample collection

A total of 40311 (ranging from 18 to 75 years) women who participated in cervical cancer screening were included continuously from August 2009 to April 2014 by the laboratory. The participants were enrolled according to the following selection criteria: non-pregnant, sexually active, without miscarriage in past 6 months. Moreover, we excluded participants who have a history of total hysterectomy, or were being treated with vaginal medicine at that time or will have surgery for cervical diseases in 6 months. Sexual activity was defined as any sexual intercourse that included vaginal penetration. Girls or adolescents with no history of sexual activity, i.e. vaginal (penile, digital or oral), anal or oral intercourse, were considered not sexually active. All women had undergone cervical cancer screening in our Health Check Center or at the Department of Obstetrics and Gynecology, The First Affiliated Hospital of Chongqing Medical University. All cervical samples of the participants were gained by professional gynecologists via cytobrush and resuspended in 20 ml of liquid-based cytology medium (Tellgen Life Science Co.Ltd.). Cervical samples were continuously accessed for cervical cytology and the existence of HPV DNA.

### DNA extraction

In short, 50 μl of the liquid-based cytology sample was moved to Eppendorf tube. The Eppendorf tube was centrifuged and the supernatant was discarded. Then, 200 μl of the denaturing reagent (Tellgen Life Science Co. Ltd. Shanghai, China) was added in the Eppendorf tube to extract genomic DNA. Then the tube was incubated at 100 °C for 10 min. The solution was centrifuged and the supernatant was moved to another Eppendorf tube. The nucleic acid concentration was calculated by examining the A260/A280 ratio (1.8–2.0).

### PCR amplification

Cervical exfoliated specimens were examined for HPV DNA using Tellgen plex™ xMAP™ HPV DNA Test assay (Tellgen Life Science Co. Ltd.). The following HPV types were detected by the assay: low-risk HPV (LR-HPV): HPV6, 11, 40/42/44, and 61/73; high-risk HPV (HR-HPV):HPV16, 18, 31, 33, 35, 39, 45, 51, 52, 53, 56, 58, 59, 66, 68, 82, 83 and 26/55.

On the basis of the manufacturer’s instructions, PCR was performed in 20 μl reaction mixture per person containing 100 ng of DNA, 10 μl PCR premixed solution, 0.8 μl thermus aquaticus (Taq) and 5 μl primer mixture. Amplification was carried out by initial denaturation at 95 °C for 5 min followed by 5 cycles of denaturation at 95 °C for 30s, annealing at 58 °C for 30s and elongation at 72 °C for 30s. In total, 40 cycles were conducted.

### Multi-analyte suspension array application

Twenty-two microliters of hybridization reagent (containing 5000 microspheres labeled with every type-specific probe) and 3 μl of the denatured biotin-labeled PCR solution were added into 96-wellmicrotiterplates (Tellgen Life Science Co. Ltd.) for every well. The mixture was incubated in 99 °C for 5 min and maintained at 48 °C for 30 min for hybridization. Seventy-five microliters of Streptavidin-R-Phycoerythrin was added into the plates after hybridization. Reactions were performed at 48 °C for 15 min. The last, the mixture was tested by a Luminex 200 analyzer instrument (Luminex Corporation, Austin, TX).

### Cytological diagnosis criteria

Cervical slides were prepared using a liquid-based cytology method, the ThinPrep system (Cytyc Corporation). Cytological classifications of disease grade were made in conformity to the Bethesda 2001 criteria. The slides were evaluated for cervical cytology by three academic cytopathologists of the First Affiliated Hospital of Chongqing Medical University. A diagnosis was assigned to each case as having negative for intraepithelial lesion or malignancy (NILM) or having an epithelial cell abnormality such as atypical squamous cells of undetermined significance (ASCUS), low-grade squamous intraepithelial lesion (LSIL), high-grade squamous intra epithelial lesion (HSIL) and invasive cervical cancer (ICC).

### Histological (biopsy) samples

Women underwent colposcopy with the Preventive Oncology International, Inc. (POI) micro-biopsy protocol of directed and random biopsies if they met one of the following criteria: (1) had LSIL or greater diagnoses on cytology; (2) were from a rural site testing positive for HPV infection by HC2 by either self or direct collection; or (3) were from an urban site and had ASC-US/HPV-positive or LSIL or greater cytological findings.

### Histological diagnosis and colposcopy

The results had been obtained by three academic Gynecology doctors of the First Affiliated Hospital of Chongqing Medical University. A diagnosis was assigned to each case as having NO Lesion, cervical intraepithelial neoplasia grade 1 (CIN 1), CIN 2, CIN 3.

### Statistical analysis

All analyses were carried out using SPSS 17.0 statistical software. Type-specific prevalence of HPV infection and their exact binomial 95% confidence intervals (CI) were calculated for women overall and stratified by age as well as by cytologic and histologic findings. Pearson’s *χ*2 test was performed to evaluate the significance of differences between designated groups. All analyses were two-sided and interpreted as being significant at *P* ≤ 0.05.

## Results

### Age-specific prevalence of HPV in the study population

In all, 40311 women were recruited HPV screening in Chongqing during 2009–2014. And 10542 (26.2%, 10542/40311) were positive for HPV. The average age of the study population was 46.2 years (range 18–75 years). The prevalence of HPV exhibited reached a peak at 31–40 years and 41–50 years, with a positive proportion of 9.4 and 8.0%, respectively (Table 1).

**Table 1:** Age-specific results of HPV genetyping in 40311 women

HPV screening						
Age group (y)	Total		Negative		Positive	
	No	%^a^	No	% ^a^	No	% ^a^
≤20	429	1.1	264	0.7	165	0.4
21–30	9970	24.7	7537	18.7	2433	6.0
31–40	14604	36.2	10811	26.8	3793	9.4
41–50	12542	31.1	9315	23.1	3227	8.0
51–60	1975	4.9	1360	3.4	615	1.5
61–70	614	1.5	390	0.9	224	0.6
≥71	177	0.4	92	0.2	85	0.2
Total	40311	100	29769	73.8	10542	26.2

*HPV* human papillomavirus

^a^Percentage of all women in the age group

### The potential risk factors in relation to HPV infection

Some potential risk factors in related to overall HPV infection were also examined. Most of the 40311 women had no smoking habit (94.0%), 25.9% of these participants were HPV-positive. The HPV infection ratio of the current smoker was 28.8%. As shown in Table 2, 22.0% of 40311 women reported that they experienced first intercourse before or at age of 20 years, the risk of HPV infection (25.9%) was no difference with other groups (26.5 and 25.7%). Women having three or more sexual partners had significantly higher risk of HPV infection (32.3%).

**Table 2:** The potential risk factors in relation to HPV infection in 40311 women

Characteristics		No. of case (%)(*N*)	No. of HPV+(*n*)	*n*/*N*	%(95%CI)
Smoking	Never	37876 (94.0)	9867	9845/37876	25.9 (23.1–29.2)
	Former	1385 (3.4)	394	394/1385	28.4 (25.3–32.6)
	Current	1050 (2.6)	281	303/1050	28.8 (26.1–31.3)
The age at first sex (y)	≤20	8884 (22.0)	2307	2307/8884	25.9 (23.3–27.2)
	21–25	18768 (46.6)	4979	4979/18768	26.5 (22.1–27.4)
	≥26	12659 (31.4)	3256	3256/12659	25.7 (23.1–28.1)
The number of sex partners	1	28960 (71.8)	7071	7271/28960	25.1 (22.4–27.9)
	2	7659 (19.0)	2076	2076/7659	27.1 (24.3–30.4)
	≥3	3692 (9.2)	1395	1195/3692	32.3 (34.1–40.2)

### HPV genotype distribution in the 10542 HPV-positive population

Twenty-five different HPV types were identified in the study population. There were 20 high-risk HPV genotypes (HPV16, 18, 26, 31, 33, 35, 39, 42, 45, 51, 52, 53, 55, 56, 58, 59, 66, 68, 82 and 83) and five low-risk genotypes (HPV6, 11,43, 44 and 61) (Table 3). The six most prevalent genotypes were HPV16 (29.9%), HPV52 (18.6%), HPV58 (17.1%), HPV18 (10.1%), HPV33 (7.53%) and HPV83 (6.9%).

**Table 3:** HPV genotype distribution in the 10542 HPV-positive population

	Single(*n*)	Multiple(n)	Total(*n*)	%	95%CI
High Risk					
16	2476	675	3151	29.89	29.02–30.07
18	785	281	1066	10.11	9.54–10.59
26	1	0	1	0.01	0.01–0.03
31	232	147	379	3.60	3.24–3.95
33	492	302	794	7.53	7.03–8.04
35	54	4	58	0.55	0.41–0.69
39	188	170	357	3.39	3.04–3.73
42	45	5	50	0.47	0.34–0.61
45	115	24	139	1.32	1.10–1.54
51	47	48	95	0.90	0.72–1.08
52	1413	545	1958	18.57	17.83–19.32
53	811	82	893	8.47	7.94–9.00
55	0	0	0	0	0
56	178	57	235	2.23	1.95–2.51
58	1372	430	1802	17.09	16.37–17.81
59	107	182	289	2.74	2.43–3.05
66	1	0	1	0.01	−0.01–0.03
68	301	170	471	4.47	4.07–4.86
82	25	0	23	0.22	0.13–0.31
83	648	80	728	6.91	6.42–7.39
Low Risk					
6	402	31	433	4.11	3.73–4.49
11	416	42	458	4.34	3.96–4.73
44	139	16	155	1.47	1.24–1.70
43	1	0	1	0.01	−0.01 0.03
61	130	11	130	1.23	1.02–1.44
Group1/2A			10794	79.88	79.21–80.56
Group B			918	6.79	6.37–7.22
Group 3			1800	13.22	12.15–13.89

The HPV genotypes were detected in 10542 HPV-positive with the Luminex Array. High-risk types include 20 oncogenic types and the other 5 types are classified intolow types. Single is when one type was detected as the sole type. Multiple is when >1 type is detected. Several samples had more than one HPV type detectable by PCR, thus the sum of the number of positive cases for each type of HPV exceeds the total number of samples in cases of multiple type infections

Group1/2A type (16, 18, 31, 33, 35, 39, 45, 51, 52, 56, 58, 59 and 68), coinfection with HPV types of Group 2B and/or Group 3 allowed

Group 2B HPV type (26, 30, 53, 66, 67, 69, 70, 73 and 82), coinfection with HPV types of Group 3 allowed

Group 3 HPV type (6, 7, 9, 11, 40, 42, 43, 61, 74, 81, 83, 86, 87, 89, 90, 91 and 114)

Among HPV positives amples, 79.88% contained HPV genotypes belonging to carcinogenic Group 1/2A. Further, 6.79% of positive samples contained possibly carcinogenic HPV types (Group 2B) and 13.22% low-risk types (Group 3).

In order to analyze the correlation between prevalent genotypes of HPV infection and cervical lesions, using the selection criteria (as Method part shown), a part of women were chosen for cervical biopsy or colposcopy as mentioned above.

### HPV genotype distribution according to colposcopy diagnostics

Among the 10542 HPV-positive women, 493 had colposcopy data. The estimated attribution for each cytological grade is shown in Table 4. The most common HPV genotypes were HPV16, 52 and 18 in cervical intraepithelial neoplasia (CIN) 1; HPV52, 16, 58, 33 and 18 in CIN 2; and HPV52, 16, 58, 33 and 18 in CIN 3. A large majority of the Group 1/2A HPV types accounted for 84.5, 85, 97.2, and 97.9% in NO lesion, CIN1, CIN2, CIN3, respectively. The prevalence of Group 2B was 5, 4.8, 1.4, and 0.9% in NO lesion, CIN1, CIN2, CIN3, respectively. The proportion of infections with Group 3 was 10.5, 10.0, 1.4, 1.3% in NO lesion, CIN1, CIN2, CIN3, respectively.

**Table 4:** HPV genotype distribution according to colposcopy diagnostics

Type	NO lesion (*n* = 208)			CIN I (*n* = 19)			CIN 2 (*n* = 57)			CIN 3 (*n* = 209)		
	*n*	%	95%CI	*n*	%	95%CI	*n*	%	95%CI	*n*	%	95%CI
High risk												
16	64	30.8	24.5–37.0	6	31.5	10.6–52.4	27	47.3	34.4–60.3	144	68.9	62.6–75.2
18	23	11.1	6.8–15.3	2	10.5	−3.2–24.3	6	10.5	2.6–18.5	15	7.2	3.7–10.7
26	0	0	0	0	0	0	0	0	0	0	0	0
31	8	3.9	1.2–6.5	0	0	0	4	7.0	0.4–13.7	8	3.8	1.2–6.4
33	13	6.3	3.0–9.5	1	5.2	−4.7–15.3	8	14.0	5.0–23.0	15	7.2	3.7–10.7
35	1	0.5	−0.5–1.4	0	0	0	0	0	0	1	0.5	−0.5–1.4
39	7	3.4	0.9–5.8	1	5.3	−4.7–15.3	1	1.7	−1.7–5.2	0	0	0
42	0	0	0	0	0	0	0	0	0	0	0	0
45	0	0	0	0	0	0	1	1.7	−1.7–5.2	1	0.5	−0.5–1.4
51	1	0.5	−0.5–1.4	0	0	0	0	0	0	0	0	0
52	30	14.4	9.7–19.2	5	26.3	6.5–46.1	8	14.0	5.0–23.0	14	6.7	3.3–10.1
53	10	4.8	1.9–7.7	1	5.3	−4.7–15.3	1	1.7	−1.7–5.2	2	1.0	−0.4–2.3
55	1	0.5	−0.5–1.4	0	0	0	0	0	0	0	0	0
56	4	1.9	0.1–3.8	1	5.3	−4.7–15.3	0	0	0	0	0	0
58	31	14.9	10.1–19.7	1	5.3	−4.7–15.3	10	17.5	7.7–27.4	24	11.5	7.2–15.8
59	5	2.4	0.3–4.5	0	0	0	2	3.51	−1.3–8.3	7	3.4	0.9–5.8
66	2	1.0	−0.4–2.3	0	0	0	0	0	0	0	0	0
68	15	7.2	3.7–10.7	0	0	0	2	3.51	−1.3–8.3	2	1.0	−0.4–2.3
82	0	0	0	0	0	0	0	0	0	0	0	0
83	9	4.3	1.6–7.1	1	5.3	−4.8–15.3	1	1.8	−1.7–5.2	2	1.0	−0.4–2.3
Low Risk												
6	6	2.9	0.6–5.2	0	0	0	0	0	0	1	0.5	−0.5–1.4
11	9	4.3	1.6–7.1	5.3	0.2	−4.8–15.3	0	0	0	0	0	0
44	4	1.9	0.1–3.8	0	0	0	0	0	0	1	0.5	−0.5–1.4
43	0	0	0	0	0	0	0	0	0	0	0	0
61	1	0.5	−0.5–1.4	0	0	0	0	0	0	0	0	0
Group1/2A	202	84.5	79.9–89.1	17	85	69.4–100.7	69	97.2	93.3–101.0	231	97.9	96.0–99.7
Group 2B	12	5.0	2.25–7.79	1	4.8	−4.6–14.6	1	1.4	−1.3–4.2	2	0.9	−0.3–2.0
Group 3	25	10.5	6.58–14.34	2	10.0	−3.2–23.2	1	1.4	−1.3–4.2	3	1.3	−0.2–2.7

### HPV genotype distribution according to histological diagnosis

As shown as Table 5, 585 of 10542 HPV-positive women were tested for cervical biopsy. The most common HPV genotypes were HPV16 in CIN 1; HPV16,18 and 52 in CIN 2; and HPV52, 16, 58, 33 and 18 in CIN 3. The prevalence of the Group 1/2A HPV types accounted for 88.2, 85.2, 96.8, and 96.1% in NO lesion, CIN1, CIN2, CIN3, respectively. However, the infections ratio of Group 2B was 1.6, 0, 2.2, and 0.5% in NO lesion, CIN1, CIN2, CIN3, respectively. The proportion of infections with Group 3 was 10.2, 14.8, 1.1, 3.5% in NO lesion, CIN1, CIN2, CIN3, respectively.

**Table 5:** HPV genotype distribution according to histological diagnosis

Histological diagnosis (*n* = 585)												
	NO lesion (*n* = 113)			CIN I (*n* = 20)			CIN 2 (*n* = 82)			CIN 3 (*n* = 370)		
Type	*n*	%	95%CI	*n*	%	95%CI	*n*	%	95%CI	*n*	%	95%CI
High risk												
16	30	26.6	18.4–34.7	10	50.0	28.1–71.9	44	53.66	42.9–64.5	263	71.08	66.5–75.7
18	15	13.3	7.0–19.5	0	0	0	3	3.66	−0.4–7.7	33	8.92	6.0–11.8
26	0	0	0	0	0	0	0	0		0	0	0
31	4	3.5	0.1–7.0	0	0	0	4	4.88	0.2–9.5	13	3.51	1.6–5.4
33	7	6.2	1.8–10.6	0	0	0	7	8.54	2.5–14.6	27	7.30	4.7–9.9
35	2	1.8	−0.7–4.2	1	5.0	−4.6–14.6	0	0	0	1	0.27	−0.3–0.8
39	3	2.7	−0.3–5.6	0	0	0	0	0	0	1	0 .27	−0.3–0.8
42	0	0	0	0	0	0	0	0	0	1	0.27	−0.3–0.8
45	0	0	0	0	0	0	2	2.44	−0.9–5.78	1	0.27	−0.3–0.8
51	0	0	0	0	0	0	0	0	0	0	0	0
52	22	19.5	12.2–26.8	6	30.0	9.9–50.1	8	9.76	3.3–16.2	18	4.86	2.7–7.1
53	4	3.5	0.1–7.0	0	0	0	2	2.44	−0.9–5.8	2	0.54	−0.2–1.3
55	1	0.9	−0.8–2.6	0	0	0	0	0	0	0	0	0
56	1	0.9	−0.8–2.6	0	0	0	2	2.44	−0.9–5.8	2	0 .54	−0.2–1.3
58	14	12.4	6.3–18.5	3	15.0	−0.7–30.7	16	19.51	10.9–28.1	42	11.35	8.1–14.6
59	3	2.7	−0.3–5.6	2	10.0	−3.2–23.1	1	1.2	−1.2–3.6	7	1.9	0.5–3.3
66	1	0.9	−0.8–2.6	0	0	0	0	0	0	0	0	0
68	11	9.7	4.3–15.2	1	5.0	−4.5–14.5	3	3.7	−0.4–7.7	5	1.4	0.2–2.5
82	0	0	0	0	0	0	0	0	0	0	0	0
83	6	5.3	1.2–9.4	0	0	0	1	1.22	−1.2–3.6	10	2.7	1.1–4.4
Low Risk												
6	1	0.9	−0.8–2.6	2	10.0	−3.2–23.2	0	0	0	2	0.5	−0.2–1.3
11	5	4.4	0.6–8.2	2	10.0	−3.2–23.2	0	0	0	1	0.3	−0.3–0.8
44	2	0.34	0	1	5.00	−4.6–14.6	0	0	0	1	0.3	−0.3–0.8
43	0	1.77	−0.7–4.2	0	0	0	0	0	0	0	0	0
61	1	0.9	−0.8–2.6	0	0	0	0	0	0	0	0.3	−0.3–0.8
Group1/2A	112	88.2	82.6–93.8	23	85.2	71.8–98.6	90	96.8	93.2–100.4	413	96.1	94.2–97.9
Group2 B	5	1.6	−0.6–3.7	0	0	0	2	2.2	−0.8–5.1	2	0.5	−0.2–1.1
Group 3	13	10.2	5.0–15.5	4	14.8	1.4–28.2	1	1.1	−1.0–3.2	15	3.5	1.8–5.2

HPV genotypes were detected with the Luminex Array. Single is when one type was detected as the sole type. Multiple is when >1 type is detected. Several samples had more than one HPV type detectable by PCR, thus the sum of the number of positive cases for each type of HPV exceeds the total number of samples in cases of multiple type infections. Cervical abnormalities included ASCUS, LSIL, HSIL and ICC

The estimated attribution for each cytological grade is shown in Table 6. Single-type infections were detected in 75.1% (109/145) in ASCUS, 20.0% (1/5) in LSIL, 72.6% (61/84) in HSIL, and 78.3% (29/37) in invasive cervical cancer (ICC). Furthermore, single high-risk HPV genotypes infections were substantially more frequent (68.3%) than single low-risk genotypes infections (6.8%) in ASCUS. There were high-risk and low-risk HPV genotypes infections in ASCUS. However, there are almost high-risk HPV genotypes infections in LSIL, HSIL and ICC. Multiple-type infections were 24.9% (36/145) in ASCUS, 80.0 (4/5) in LSIL, 27.4% (23/84) in HSIL and 21.7% (8/37) in ICC. The most common HPV genotypes were HPV16, 52, 18 and 58 in ASCUS; HPV16 in LSIL; HPV16 and 58 in HSIL; and HPV16 in ICC.

**Table 6:** HPV genotype distribution according to cytology abnormalities

	ASCUS (*n* = 145)			LSIL (*n* = 5)				HSIL (*n* = 84)		ICC (*n* = 37)		
HPV infection	Single	Multiple	Total	Single	Multiple	Total	Single	Multiple	Total	Single	Multiple	Total
	*n*(%)	*n*(%)	*n*(%)	*n*(%)	*n*(%)	*n*(%)	*n*(%)	*n*(%)	*n*(%)	*n*(%)	*n*(%)	*n*(%)
High-Risk	99(68.3)	34(23.4)	134(92.4)	1(20)	4(80)	5(100)	61(72.6)	23(27.4)	84(100)	29(90.1)	8(9.1)	37(100)
Low-Risk	10(6.8)	1(0.6)	11(7.6)	0(0)	0(0)	0(0)	0(0)	0(0)	0(0)	0(0)	0(0)	0(0)
High risk type												
16	36(24.8)	10(6.9)	46(31.7)	0	2(40)	2(40)	32(38.1)	10(11.9)	42(50.0)	18(48.7)	3(8.1)	21(56.8)
18	11(7.5)	2(1.4)	13(8.9)	1(20)	1(20)	2(40)	0(0)	0(0)	0(0)	4(10.8)	0(0)	4(10.8)
26	0(0)	0(0)	0(0)	0(0)	0(0)	0(0)	0(0)	0(0)	0(0)	0(0)	0(0)	0(0)
31	4(2.8)	0(0)	4(2.8)	0(0)	0(0)	0(0)	3(11.9)	1(1.1)	4(13.0)	0(0)	0(0)	0(0)
33	5(3.4)	2(1.4)	7(4.8)	0(0)	0(0)	0(0)	4(4.8)	3(3.6)	7(8.4)	0(0)	2(5.4)	2(5.4)
35	1(0.7)	0(0)	1(0.7)	0(0)	0(0)	0(0)	0(0)	0(0)	0(0)	0(0)	0(0)	0(0)
39	4(2.8)	1(0.7)	1(3.5)	0(0)	0(0)	0(0)	0(0)	0(0)	0(0)	0(0)	0(0)	0(0)
42	0(0)	0(0)	0(0)	0(0)	0(0)	0(0)	0(0)	0(0)	0(0)	0(0)	0(0)	0(0)
45	1(0.7)	0(0)	1(0.7)	0(0)	0(0)	0(0)	1(1.1)	1(1.1)	2(2.2)	0(0)	0(0)	0(0)
51	0(0)	0(0)	0(0)	0(0)	0(0)	0(0)	0(0)	0(0)	0(0)	0(0)	0(0)	0(0)
52	6(4.1)	6(4.1)	12(8.2)	0(0)	0(0)	0(0)	2(2.2)	3(3.5)	5(5.8)	1(2.7)	0(0)	1(2.7)
53	7(4.8)	0(0)	7(4.8)	0(0)	0(0)	0(0)	2(2.2)	0(0)	2(2.2)	0(0)	0(0)	0(0)
55	0(0)	0(0)	0(0)	0(0)	0(0)	0(0)	0(0)	0(0)	0(0)	0(0)	0(0)	0(0)
56	0(0)	3(2.1)	3(2.1)	0(0)	0(0)	0(0)	1(1.1)	0(0)	1(1.1)	0(0)	0(0)	0(0)
58	10(6.9)	6(4.1)	16(11.0)	0(0)	1(20)	1(20)	9(10.7)	3(3.6)	12(14.3)	5(13.7)	0(0)	5(13.7)
59	3(2.1)	1(0.7)	4(2.8)	0(0)	0(0))	0(0)	0(0)	0(0)	0(0)	0(0)	1(2.7)	1(2.7)
66	1(0.7)	0(0)	1(0.7)	0(0)	0(0)	0(0)	0(0)	0(0)	0(0)	1(2.7)	0(0)	1(2.7)
68	5(3.4)	3(2.1)	8(5.5)	0(0)	0(0)	0(0)	0(0)	0(0)	0(0)	0(0)	2(5.4)	2(5.4)
82	0(0)	0(0)	0(0)	0(0)	0(0)	0(0)	0(0)	0(0)	0(0)	0(0)	0(0)	0(0)
83	5(3.4)	0(0)	5(0)	0(0)	0(0)	0(0)	2(2.2)	2(2.2)	1(1.1)	0(0)	0(0)	1(2.7)
Low Risk type												
6	1(0.7)	1(0.7)	2(1.4)	0(0)	0(0)	0(0)	0(0)	0(0)	0(0)	0(0)	0(0)	0(0)
11	5(3.4)	0(0)	5(3.4)	0(0)	0(0)	0(0)	0(0)	0(0)	0(0)	0(0)	0(0)	0(0)
44	4(2.8)	0(0)	0(0)	0(0)	0(0)	0(0)	0(0)	0(0)	0(0)	0(0)	0(0)	0(0)
43	0(0)	0(0)	0(0)	0(0)	0(0)	0(0)	0(0)	0(0)	0(0)	0(0)	0(0)	0(0)
61	0(0)	0(0)	0(0)	0(0)	0(0)	0(0)	0(0)	0(0)	0(0)	0(0)	0(0)	0(0)
Group1/2A		116(80)			5(100)			73(87)			36(95)	
Group 2B		8(5.5)			0(0)			2(2.3)			2(5.2)	
Group 3A		8(5.5)			0(0)			2(2.3)			1(2.6)	

HPV genotypes were detected with the Luminex Array. Single is when one type was detected as the sole type. Multiple is when >1 type is detected. Several samples had more than one HPV type detectable by PCR, thus the sum of the number of positive cases for each type of HPV exceeds the total number of samples in cases of multiple type infections. Cervical abnormalities included ASCUS, LSIL, HSIL and ICC

A large majority of the Group 1/2A HPV types accounted for 80%, 100%, 87%, 95% in ASCUS, LSIL, HSIL, ICC, respectively. However, the infections ratio of Group 2B was 5.5%, 0, 2.3%, 5.2% in ASCUS, LSIL, HSIL, ICC, respectively. The proportion of infections with Group 3 was 5.5%, 0, 2.3%, 2.6% in ASCUS, LSIL, HSIL, ICC, respectively.

### Consistency of colposcopy, histological diagnosis and cytological diagnosis

**Fig 1:**
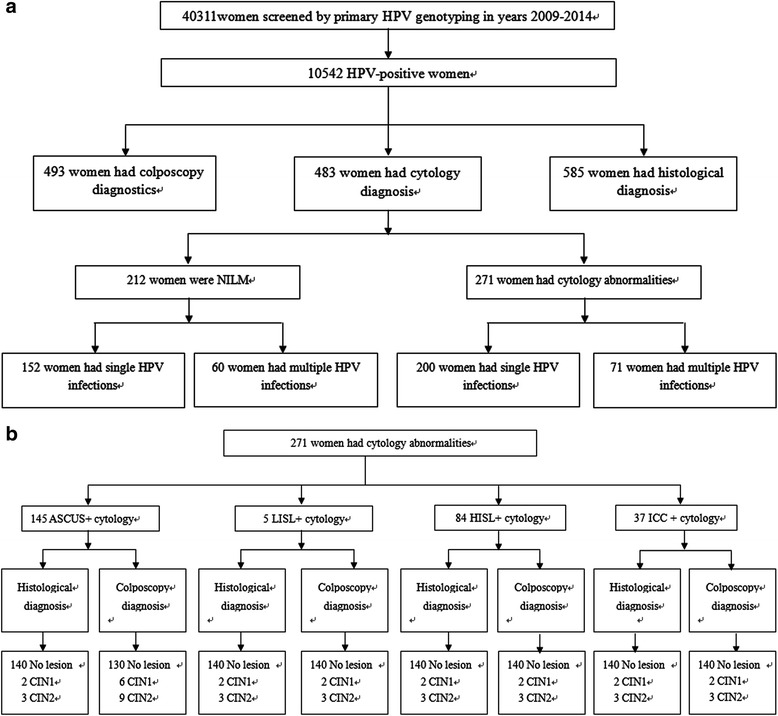
**a** Flow chart of the study samples. Prevalence of single and multiple HPV infections according to cytological diagnosis. NILM: negative for intraepithelial lesion or malignancy. The *P* values compare HPV singleinfections and multipleinfections, with a *P* < 0.05 considered significant. By Pearson’s *χ*2 test, *χ*2 = 2.592, *P* = NS. **b** The consistency of the colposcopy indicated, histological diagnosis and cytology diagnosis

Figure 1a described the process by which we obtained our final results. Among the 483 women tested for cytological diagnosis, single-type HPV infection was found in 71.6% of women with NILM compared with 73.8% of women with abnormal cytology. The prevalence of multiple HPV infections was 28.4% in NILM compared with 26.2% in abnormal cytology (Fig. 1a). However, this association was not significant (*P* = 0.207).

Among the 271 cytology abnormalities, we compared the data for colposcopy and histological diagnosis. In the ASCUS-positive group, histological diagnosis showed 140 with no lesion, two CIN 1 and three CIN 2, while colposcopy indicated 130 with no lesion, six CIN 1 and nine CIN 2. In the HSIL-positive group, histological diagnosis showed 56 CIN 1, 20 CIN 2 and eight CIN 3, while colposcopy indicated 45 CIN 1 and 39 CIN 2. However, histological diagnosis showed four CIN 1, eight CIN 2 and 25 CIN 3, while colposcopy indicated six CIN 1, nine CIN 2 and 22 CIN 3 in the ICC-positive group. The data showed that cytological diagnosis was consistent with colposcopy and histological diagnosis (Fig. 1b).

## Discussion

HPV infection is the most common sexually transmitted disease, which is connected with various clinical status, ranging from asymptomatic infection to malignant cervical disease. Our study was a large-scale report which demonstrated the type-specific HPV prevalence in Chongqing, and its relation to the cervical disease burden. The three most prevalent carcinogenic HPV types in the Chongqing screening population were HPV16 (0.7%), 52 (0.5%) and 58 (0.4%). The prevalence of HPV16 in our study was slightly lower than that reported in the women with normal cytology of Western and Northern European countries [13, 14]. Regardless of cervical cytology, HPV16 was the most prevalent genotype reported worldwide, followed by HPV42, 58, 31, 18 and 56 in histological specimens of precancerous lesions [15]. HPV16 in cervical cytology was twice as frequent as any other high-risk type in all regions and the next most common high-risk types were HPV33 and HPV56 in Asia, HPV58 in South America, and HPV31 in Europe in general population. Sub-Saharan Africa has the highest prevalence of all HPV types in general population and Europe the lowest [16]. HPV 52 infection among women with normal cytology ranks second in Africa and third in Denmark, but is not the most frequent genotypes for most of Europe [16–18]. In China, HPV16/18 was the predominant types, followed by HPV58, 31 and 52 in histological samples of cancer [17, 18]. We detected HPV16, 52 and 58 more frequently than HPV18, which was notably consistent with the data of Bao et al.[19].

HPV 18 ranks second genotype in Europe about general population [19], but showed a lower prevalence in our study. It has been demonstrated that HPV16 is associated with both squamous cell carcinoma and adenocarcinoma, whereas HPV18 is mainly a risk factor for development of adenocarcinoma [20]. The type of cervical cancer mostly was squamous cell carcinoma in the study, so the prevalence of HPV18 was lower than other studies [19].

High-risk HPV infection is the principal factor in cervical cancer. Distribution of HPV genotypes showed increased prevalence of high-risk HPV in direct relation to the severity of cervical cytopathology [21]. In the present study, according to cytology abnormalities, the prevalence of high-risk HPV was 92.0, 100, 100, 100 and 100% in ASCUS, LSIL, HSIL and ICC, respectively. When cytobrush was used to test high-risk HPV in ICC, positivity rates were between 90 and 100% in all patients [22]. Therefore, the infection of high-risk HPV genotypes is consisted with a risk of disease progression. In our study, HPV 16 and 18 accounted for 68% of ICC, which was consistent with the prevalence worldwide.

Among the population with CIN 2 and 3, HPV 16, 31, 33, 18 and 58 were the most common genotypes [22]. In Chongqing, the most prevalent HPV types in CIN 3+ lesions were HPV 16, 58 and 18. A worldwide research of 10575 cases of ICC indicated that HPV-DNA-based screening should focus on HPV16 and 18 [11]. New study demonstrated that HPV16 and 18 are about equally important in ADC [14, 23]. HPV prevalence ranged from 9.3% in mainland China to 15.0% in Taiwan in NILM [16]. This research (44.9%, 217/483) was much higher than the early reports. It might be due to people who came to the hospital with an unhealthy or disease states. In a large-scale Chinese research, the most frequent HPV-DNA-based genotypes were 16, 58, 52, 18, 39, 33, 68, 31, 66 and 6 in women with normal diagnoses [19, 24], which consisted with our study. The reason of female patients with a negative cytology data but HPV-positive was low viral load in the early periods of HPV infection. Usually, most HPV infections are eliminated by cell-mediated immune system within 6 months. However, some high-risk HPV genotypes infections may take longer time to be cleared away, frequently between 12 and 24 months [25].

The age peak of HPV prevalence was 31–40 years, which was different from 45 years onwards reported in Latin America, the Caribbean, and most countries across Africa [17]. A peak at <25 years of age was reported in Finland [26]. Sexual behavior, viral characteristics, and host susceptibility may account for the prevalence differences.

The results regarding the effect of smoking on HPV infection have been contradictory. Some studies revealed that smoking was associated with an increased trend of HPV prevalence [27], but not others [28]. Our results demonstrated an increased prevalence of HPV in current smokers compared with non-smokers. But the numbers of current and former smokers in our study were not sufficiently large to draw a conclusion.

In the present study, our results showed that women having three or more sexual partners had significantly higher risk of HPV infection. The results was consisted with other studies [29], revealing that the sexual behavior is an important determinant of HPV infection. The more sexual partners that a woman has, the higher risk of getting HPV infected

Similar to other studies [24], the prevalence of HPV16 and Group1/2A HPV types increased with CIN grade. Low-grade cervical lesions can also be caused by low-risk HPV types and are merely a manifestation of viral infection.

This is the first study to identify Initial impact of HPV infection in non-vaccinated women of Chongqing. We report the results of a population-based HPV screening of 40311 women in Chongqing Province of China. Our studies would demonstrate the understanding of the epidemiology of HPV infection. The data will assist in developing a putative screening program, that the HPV types would be included in the HPV vaccine in future to provide protection against those high-risk types of HPV with high prevalence identified in our study. However, there are several limitations in this study, one of which is the absence of some risk factors, such as gravidity, number of abortion, condom use and education. And a population-based research about the correlation between these risk factors with cervical cancer will be the direction of our further study. Moreover, larger and prospective studies about the mechanism are also needed to validate our findings.

## Conclusion

Our results give a broad summary of the distribution of HPV type in Chongqing Province in women with different grades of cervical lesions. The most common HPV types were 16, 52, 58 and 18. Further, large, multicenter prospective studies are needed to fully evaluate the varied HPV pattern in Chinese women, and to provide substantial information directly useful for planning HPV preventive vaccination in China.

## Abbreviations

ASCUS: Atypical squamous cells of undetermined significance
CI: Confidence intervals
CIN: Cervical intraepithelial neoplasia grade
HPV: Human papillomavirus
HSIL: High-grade squamous intra epithelial lesion
ICC: Invasive cervical cancer
LSIL: Low-grade squamous intraepithelial lesion
NILM: Negative for intraepithelial lesion or malignancy
Pap: Papanicolaou
